# Comparative characterization of a novel cad-cam polymer-infiltrated-ceramic-network

**DOI:** 10.4317/jced.52521

**Published:** 2015-10-01

**Authors:** Alberto Albero, Agustín Pascual, Isabel Camps, María Grau-Benitez

**Affiliations:** 1 Associate Professor, Department of Dental Materials, European University of Valencia; 2 Assistant Professor, Department of Dental Materials, University of Valencia; 3 Associate Professor, Department of Dental Materials, University of Valencia

## Abstract

**Background:**

The field of dental ceramics for CAD-CAM is enriched with a new innovative material composition having a porous three-dimensional structure of feldspathic ceramic infiltrated with acrylic resins.The aim of this study is to determine the mechanical properties of Polymer-Infiltrated-Ceramic-Network (PICN) and compare its performance with other ceramics and a nano-ceramic resin available for CAD-CAM systems.

**Material and Methods:**

In this study a total of five different materials for CAD-CAM were investigated. A polymer-infiltrated ceramic (Vita Enamic), a nano-ceramic resin (Lava Ultimate), a feldspathic ceramic (Mark II), a lithium disilicate ceramic (IPS-e max CAD) and finally a Leucite based ceramic (Empress - CAD). From CAD-CAM blocks, 120 bars (30 for each material cited above) were cut to measure the flexural strength with a three-point-bending test. Strain at failure, fracture stress and Weibull modulus was calculated. Vickers hardness of each material was also measured.

**Results:**

IPS-EMAX presents mechanical properties significantly better from the other materials studied. Its strain at failure, flexural strength and hardness exhibited significantly higher values in comparison with the others. VITA ENAMIC and LAVA ULTIMATE stand out as the next most resistant materials.

**Conclusions:**

The flexural strength, elastic modulus similar to a tooth as well as having less hardness than ceramics make PICN materials an option to consider as a restorative material.

** Key words:**Ceramic infiltrated with resin, CAD-CAM, Weibull modulus, flexural strength, micro hardness.

## Introduction

One of the main objectives of restorative dentistry is to replace lost tooth structure with a material whose structure and physical properties are similar to a natural tooth (1). For this purpose, CAD-CAM technology is rapidly becoming popular, as it reduces the number of clinical sessions and manufacturing time of indirect restorations. Furthermore, the CAD-CAM system allows the use of new materials with improved properties compared with other materials used in direct restorative procedures (2).

Ceramics, because of their chemical stability, have good mechanical and optical properties, as well as excellent biocompatibility. However, once placed in the mouth, repairs are often problematic if they are necessary. In contrast, the composites are easier to operate and repair but its wear, biocompatibility and mechanical properties are inferior to ceramic (3). Therefore, some authors suggest associating the elastic modulus of composites, which is similar to dentin, with feldspathic ceramic, similar to enamel, that would add long-term aesthetics, looking to make the ideal restorative material (4). With the intention of achieving this goal easily, a new material has recently been developed that attempts to emulate the properties of a natural tooth that is called polymers-infiltrated-ceramic-network (PICN) (1,3-9). PICN consist of two interlocking phases, a porous sinterized feldspathic ceramic and an infiltrating polymer (for dental use commonly methacrylates) (1).

The aim of this study is to determine the mechanical properties of polymers-infiltrated-ceramic-network (PICN) and compare its behaviour with other materials available for CAD-CAM systems. For this purpose, flexural strength, fracture load, Vickers hardness and Weibull modulus were studied.

## Material and Methods

In this study a total of five ceramic materials for CAD-CAM were investigated. Among them a polymer-infiltrated-ceramic-network (Enamic Vita, Vita Zahnfabrik, Bad Säckingen, Germany), a nano filled composite resin (Lava Ultimate, 3M ESPE, Neuss, Minn), a feldspathic ceramic (Mark II, Vita Zahnfabrik, Bad Säckingen, Germany), a lithium disilicate ceramic (IPS-e max CAD, Ivoclar Vivadent, Schaan, Liechtenstein) and finally, a Leucite based ceramic (Empress - CAD, Ivoclar Vivadent, Liechtenstein Schaab).

2.1 Flexural strength, fracture load and Weibull modulus

**Figure 1:**
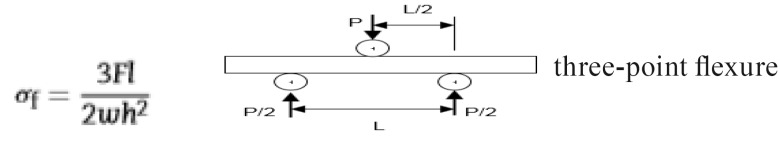
Fracture stress.

From commercialized CAD-CAM blocks, 120 bending bars (n = 30) of each material (14 mm x 4 mm x 3 mm) (ISO 6872 (ISO 2009 [10])) were cut using a cutting machine (Struers Minitom, Willich, Germany ) at a rate of 250 rpm under water irrigation, to measure flexural strength in a three point bending test. IPS- e max CAD bars were crystallized in a ceramic furnace (Programat P300, Ivoclar Vivadent, Schaan Liechtenstein) following the manufacturer’s instructions and recommendations. Subsequently, the samples were polished with abrasive discs SiC paper 500, 1200 and 2400 # (LaboPol-1, Struers, Willich, Germany). All the bending bars were chamfered in order to minimize stress concentration due to machining flaws (ISO 6872 (ISO 2009 [10])). The bars were then subjected to an increasing load until fracture in a universal testing machine (Instron 4411, Massachusetts) in a three-point flexure with a crosshead speed of 0.5 mm / min. The fracture stress was calculated using the formula (1.6): (Fig. 1).

Where F is the fracture load, L the roller span (12mm), w the width and h the height of the bar.

**Figure 2:**
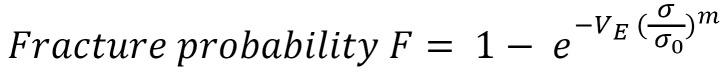
Probability of fracture.

The probability of fracture of different materials was studied using the Weibull cumulative distribution function. Values were analysed with the following formula: (Fig. 2).

Where σ is the fracture stress, VE the effective volume, σ0 the characteristic strength (the strength occurring at a 63,2% probability of failure) and m the Weibull modulus.

2.2. Vickers hardness.

**Figure 3:**
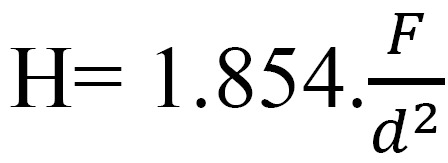
Hardness.

For each material, twenty Vickers indentations were performed with loads of 50 N with a universal testing machine (HMV Shimadzu, Kyoto, Japan). The load was maintained for 20 seconds. The resultant diagonal of the indentation and the cracks derived from the diagonals were measured with a microscope. The hardness was calculated with the formula (1), (Fig. 3).

Where F is the load and d is the indentation diagonal length.

-Specifying statistical procedures used

A general linear model (GLM) type ANOVA was estimated to compare homogeneity of average flexural strength in different materials (1,6). Levene’s test was used to test the homogeneity of variances, and if necessary, a Box-Cox transformation type is considered. Bonferroni multiple comparisons were employed to determine which pairs of materials exhibit similar average resistance with proper control of the statistical error of type I. The same GLM methodology was replicated to analyse strength and hardness.

Models for the probability of fracture of the different ceramic materials using the Weibull cumulative distribution were estimated. The estimates are based on the Griffith formula for fragile materials and they consist of obtaining characteristic force values and Weibull modulus that best fit the experimental data. Graphical representations of the probability curve based on force were per-formed to allow comparison between ceramic types. Reference significance level is 5% (α = 0.05).

## Results

3.1 Fracture load

The fracture load is highest for IPS-EMAX, with an average of 0.44 ± 0.10 kN. VITA ENAMIC, LAVA ULTIMATE L, EMPRESS and MARKII have similar mean values to each other, and a lower level with a range from 0.22 to 0.26 kN. For example, for VITA-ENAMIC materials, 50% of the pieces were broken with a force between 0.22 and 0.26 kN approximately. Moreover, half of the pieces resisted less than 0.24 kN (median). There were two atypical cases with higher strengths than usual and one sample withstood extreme load, compared to their group ( Table 1).

**Table 1:**
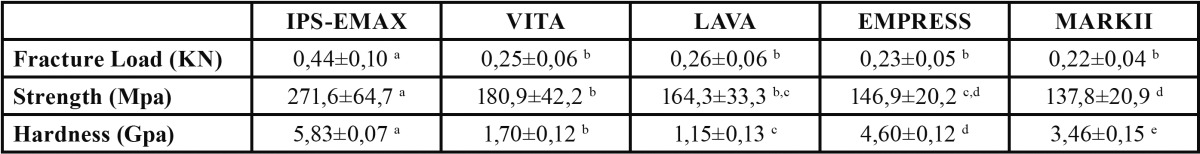
Fracture load, flexural strength and hardness of the different materials: Mean ± standard deviation. Groups with similar mean in one parameter according Bonferroni test share letter ( significance level 5%).

3.2 Flexural strength

**Figure 4:**
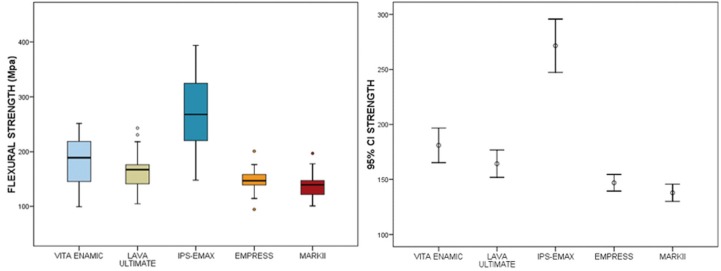
The mean and confidence intervals 95% are shown. As well as for loading, heterogeneity of variances (*p* < 0.001) was detected, so a Box- Cox Resistance - 1.27 transformation has been used for the analytical study.

The maximum mean value of the resistance is recorded in the IPS-EMAX group (271.6 ± 64.7 Mpa), far ahead of VITA EN-AMIC, ULTIMATE LAVA, EMPRESS and MARKII, which are located at a second level, with a progressive decrease in resistance. VITA ENAMIC and LAVA ULTIMATE have similar resistance; but the latter is significantly less resistant than VITA ENAMIC (*p* = 0.008). At the same time, EMPRESS is comparable to MARKII; but the latter resists significantly less than LAVA ULTIMATE (*p* <0.001) ( Table 1, Fig. 4).

The general linear model analysis of variance concluded that there are statistically significant differences (*p* <0.001) in mean values of the flexural strength of the different types of materials. Bonferroni tests allow the possibility to identify between which groups the differences are manifested ( Table 2).

**Table 2:**
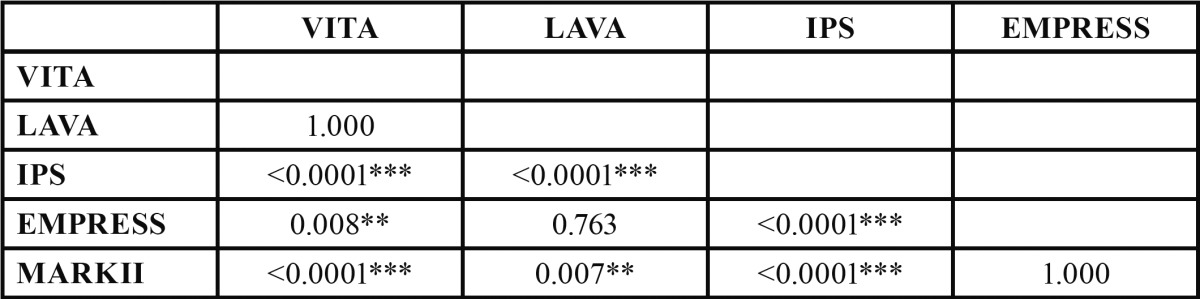
Bonferroni’s test allow to identify between wich groups the significant differences are given.

3.3 Hardness

**Figure 5:**
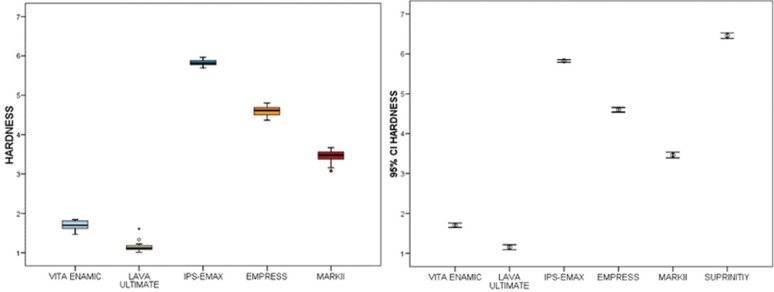
In this case, the equality of variances can be accepted from Levene test ( p> 0.05 ) and no exponential transformation is necessary.

The average hardness is highest in the IPS-EMAX group (5.83 ± 0.07) followed by EMPRESS (4.60 ± 0.12) and MarkII (3.46 ± 0.15). At a substantially lower level are the values for VITA ENAMIC and LAVA ULTIMATE ( Table 1). It is evident that each material represents a very specific and heterogeneous hardness level, compared to any other (Fig. 5).

The general linear model analysis of variance concluded that there are statistically significant differences (*p* <0.001) in mean hardness of the different types of materials. Bonferroni tests support the conclusion that each material has a significantly different hardness from the others ( Table 3).

**Table 3:**
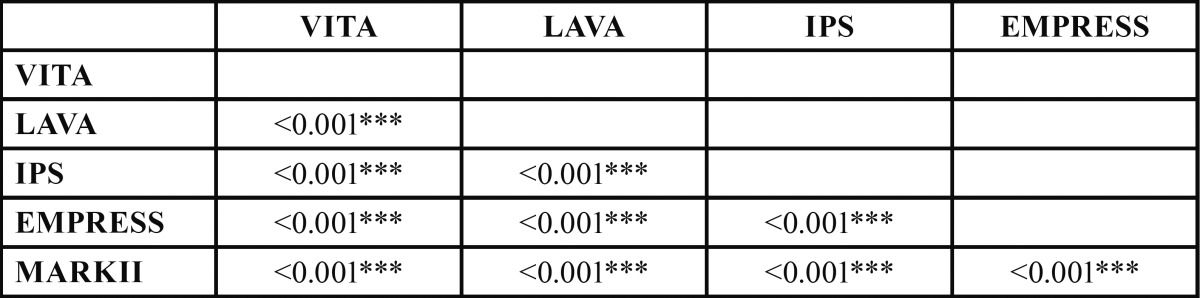
Bonferroni’s test for hardness. Can be seen that any material have significantly different hardness to any other.

3.4 Prediction of fracture probability (Weibull modulus)

The probability function of fracture is estimated for each material. To do this, the Weibull modulus and characteristic stress of the materials studied were calculated ( Table 4). The greater the characteristic force, the more resistant the material is; and the higher the Weibull modulus, the greater the impact of force on fracture probability, which is the same when the force is increased a little, the stress rises considerably and fracture probability is triggered.

**Table 4:**
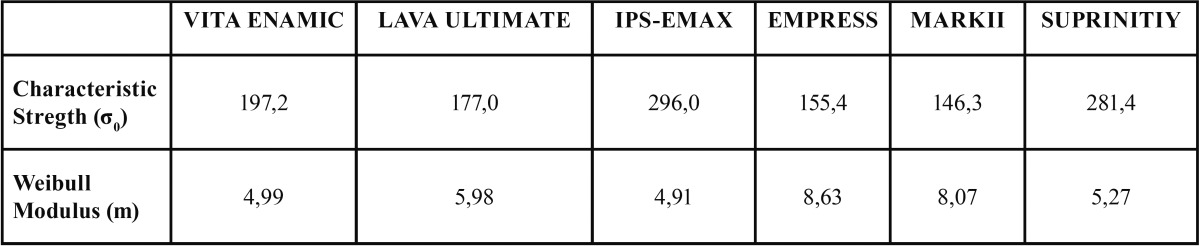
Characteristic stress and Weibull modulus of studied materials.

**Figure 6:**
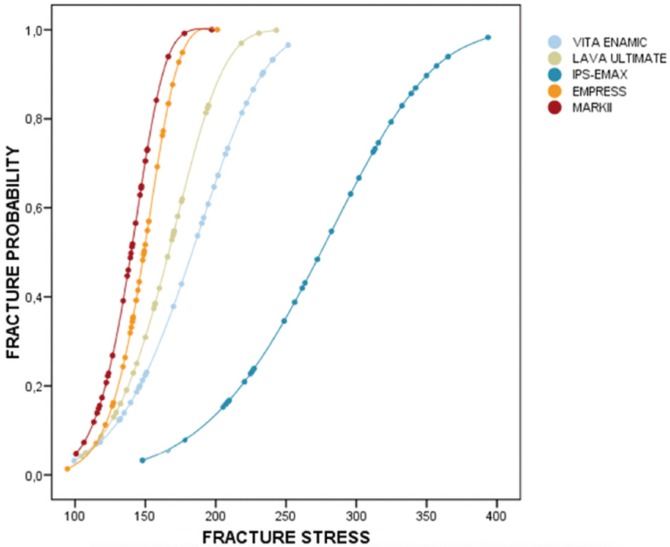
Estimation of fracture probability.

The probability curve of IPS-EMAX is very different from the rest (Fig. 6). It is shifted to the right, that is a greater force is required to have the same probability of fracture than the other materials (noted in the table above that the characteristic force is higher). Moreover, a fixed stress increase moderately increases the likelihood of rupture, because the slope of the curve is moderately soft. It needs a force of between 150 MPa to 400 MPa to move over the entire range of possible values of probability (0-1).

Of the other four materials, it is possible to distinguish two specific subgroups. Firstly, VITA and LAVA with more positive results in terms of resistance. Force characteristic (197 and 177 MPa) and weibull modulus (4.99 and 5.98) were estimated respectively. Secondly, EMPRESS and MARKII with lower characteristic force and very high modulus. That is, with little force the probability of failure is high and, moreover, it grows exponentially with small increases of force.

## Discussion

The main objective of this work is to study the mechanical properties of the PICN and compare them to those of other commercialized materials for CAD-CAM. The materials were chosen to cover multiple restorative possibilities. Currently numerous articles speak of PICN (1,3-9) and mostly non-commercialized prototypes formed by different densities of feldspathic ceramic infiltrated with resin are used to study their mechanical properties and compare them with commercialized materials (1,3,6-9). Alvaro Della Bona (4) and MA Bottino (5) use a commercially available PICN (Vita Enamic, Vita Zahnfabrik).

In the present study, a PICN is evaluated: Vita Enamic (Vita Zahnfabrik) in which, according to the manufacturer, 75% of its volume is feldspathic ceramic and 25% polymer; and a nano-ceramic resin Lava Ultimate (3M ESPE, Neuss) with 80% of ceramic nanoparticles, according to the manufacturer’s specifications.

He and Swain (7,9) described the mechanical properties of these materials and found that they were very similar to natural dentin and enamel. This has been the aim throughout the years for restorative materials. The hardness and elastic modulus similar to those of the dental tissues values makes this material a good choice for restoring posterior areas with inlays (5). Furthermore, Ausiello P. (11) indicates that the elastic modulus of adhesive cements is very similar to the PICN value, allowing a more uniform distribution of stress during mastication in restored teeth.

In PICN the density is closely related to the mechanical properties (7). Coldea Andrea (1) found that a minor fraction of the ceramic of PICN implies a lower elastic modulus and hardness, accompanied by an increase in flexural strength and failure load. In this same study (1), an increase in flexural strength of 9.2 to 28.8 MPa with increasing density is described. In turn, this also increases the elastic modulus from 3.30 to 54.5 GPa.

PICN have a lower hardness than the rest of materials studied. ( Table 1). VITA ENAMIC hardness (1.70 ± 0.12 GPa) has a value substantially lower than the rest of the ceramics, corresponding to the IPS-EMAX (5.83 ± 0.7 GPa ) the maximum hardness value. This hardness value is closer to LAVA ULTIMATE (1.15 ± 0.13 GPa). The dentin hardness ranges from 0.6 to 0.92 GPa and enamel between 3-5.3 GPa (1) The lower hardness value presented by this material is considered as an advantage when it comes to protecting the opposing tooth from massive wear (1.7).

Damage tolerance of the CIP is high when compared to other ceramics for CAD-CAM that usually breaks in mastication (6). He and Swain (7) found that the rate of fragility of the CIP had a value suitable for use in CAD-CAM. The extensions of the cracks coming out of the diagonals of the indentation were much longer in ceramics than in the CIP. The increased flexural strength of two phase materials compared to single phase, involves a reinforcement mechanism comparable to Travitzk *et al.*, Prielipp *et al.*, and Wegner *et al.* (12-14). In the last two studies, the porous ceramic was infiltrated by metal and their properties were analysed.

The most common method used to characterize a ceramic resistance and structural reliability is the Weibull statistical theory (15), which describes the resistance of a brittle material based on the probability of survival with a given stress value, which is a function of volume under stress, the strength characteristics (a normalized parameter that corresponds with a stress level where 63% of the specimens fail) and the Weibull modulus, which indicates the nature, severity and spread of the defects. High Weibull modulus values correspond to materials with a very uniform distribution of a lot of homogeneous defects with a smaller strength distribution. Low Weibull modulus values correspond to materials with non-uniform distribution of defects with a highly variable crack length and a wide distribution of strength (16). For dental ceramics, the Weibull modulus values in the literature range from 5-15 (16). In the present study the figures ranged from 4.99 for VITA ENAMIC to 8.63 for EMPRESS (see  Table 4). These values are similar to those presented by Carla Castiglia *et al.* (16) ranging from 5.2- to 11.7 for VM7 (Vita Zahnfabrik, Bad Säckingen, Germany) and D.Sign (Ivoclar Vivadent, Schaan, Liechtenstein) respectively. Although Empress and Mark II have lower resistance values, they have a greater Weibull modulus, which means that with less stress the probability of failure is high. This finding is important because in some situations, especially in areas of low stress, you can opt for a material with lower resistance but a higher Weibull modulus (16).

## Conclusions

- IPS-EMAX exhibits significantly higher values in fracture load, flexural strength and hardness. The Weibull modulus emphasizes the previous conclusion.

- VITA ENAMIC and LAVA ULTIMATE stand out as being the second most resistant materials, after IPS- EMAX. The hardness of these materials is significantly lower than ceramics.

- The flexural strength, the elastic modulus similar to the tooth and lower hardness of these materials make PICN an option to be considered as filling material.

## References

[1] Coldea A, Swain MV, Thiel N (2013). Mechanical properties of polymer-infiltrated-ceramic-network materials. Dental Materials.

[2] Van Noort R (2012). The future of dental devices is digital. Dental Materials.

[3] Nguyen JF, Ruse D, Phan AC, Sadoun MJ (2014). High-temperature-pressure polymerized resin-infiltrated ceramic networks. J Dent Res.

[4] Della Bona A, Corazza PH, Zhang Y (2014). Characterization of a polymer-infiltrated ceramic-network material. Dental Materials.

[5] Bottino MA, Campos F, Ramos NC, Rippe MP, Valandro LF, Melo RM (2015). Inlays made of a hybrid material: Adaptation and bond strengths. Oper Dent.

[6] Coldea A, Swain MV, Thiel N (2013). In-vitro strength degradation of dental ceramics and novel PICN material by sharp indentation. J Mech Behav Biomed Mater.

[7] He L, Swain M (2011). A novel polymer infiltrated ceramic dental material. Dent Mater.

[8] Petrini M, Ferrante M, Su B (2013). Fabrication and characterization of biomimetic ceramic/polymer composite materials for dental restoration. Dent Mater.

[9] He L, Purton D, Swain M (2011). A novel polymer inﬁltrated ceramic for dental simulation. J Mater Sci: Mater Med.

[10] (2009). Dentristry - ceramic materials.

[11] Ausiello P, Rengo S, Davidson CL, Watts DC (2004). Stress distributions in adhesively cemented ceramic and resin-composite Class II inlay restorations: a 3D-FEA study. Dent Mater.

[12] Travitzky NA (1998). Microstructure and mechanical properties of alumina/copper composites fabricated by different infiltration techniques. Mater Lett.

[13] Prielipp H, Knechtel M, Claussen N, Streiffer SK, Müllejans H, Rühle M (1995). Strength and fracture toughness of aluminum/alumina composites with interpenetrating networks. Materials Science and Engineering.

[14] Wegner LD, Gibson LJ (2001). The fracture toughness behaviour of interpenetrating phase composites. Int J Mech Sci.

[15] Quinn JB, Quinn GD (2010). A practical and systematic review of Weibull statistics for reporting strengths of dental materials. Dent Mater.

[16] Gonzaga CC, Cesar PF, Miranda Jr (2011). WG, Yoshimura HN. Slow crack growth and reliability of dental ceramics. Dent Mater.

